# Comparative Study of Planar Octahedron Molecular Structure via Eccentric Invariants

**DOI:** 10.3390/molecules28020556

**Published:** 2023-01-05

**Authors:** Zheng-Qing Chu, Haidar Ali, Didar Abdulkhaleq Ali, Muhammad Nadeem, Syed Ajaz K. Kirmani, Parvez Ali

**Affiliations:** 1 General Education Department, Anhui Xinhua University, Hefei 230000, China; 2 Department of Mathematics, Riphah International University, Faisalabad 38000, Pakistan; 3 Department of Mathematics, Faculty of Science, University of Zakho, Zakho 42002, Iraq; 4 Department of Electrical Engineering, College of Engineering, Qassim University, Unaizah 56215, Saudi Arabia; 5 Department of Mechanical Engineering, College of Engineering, Qassim University, Unaizah 56215, Saudi Arabia

**Keywords:** eccentricity *ξ*(*p*), total eccentricity *ϑ*(*G*), average eccentricity *avec*(*G*), Zagreb eccentricity index ℵ(*G*), geometric arithmetic eccentricity *GA*_4_(*G*), atom bond connectivity eccentricity *ABC*_5_(*G*), 05C12, 05C90

## Abstract

A branch of graph theory that makes use of a molecular graph is called chemical graph theory. Chemical graph theory is used to depict a chemical molecule. A graph is connected if there is an edge between every pair of vertices. A topological index is a numerical value related to the chemical structure that claims to show a relationship between chemical structure and various physicochemical attributes, chemical reactivity, or, you could say, biological activity. In this article, we examined the topological properties of a planar octahedron network of *m* dimensions and computed the total eccentricity, average eccentricity, Zagreb eccentricity, geometric arithmetic eccentricity, and atom bond connectivity eccentricity indices, which are used to determine the distance between the vertices of a planar octahedron network.

## 1. Introduction and Preliminary Results

Topological indices are a helpful tool provided by graph theory. Computer analysis is a modern academic field that merges chemistry, mathematics, and computer technology [1,2,3,4,5]. Quantitative structure–activity (QSAR) and structure–property (QSPR) relationships are used to predict biological activities and the properties of chemical compounds. That is why they have piqued the curiosity of academics all around the world. Topological indices are gaining attraction in the world of communication chemistry because of their application in non-empirical, quantitative structure–property and quantitative structure–activity relationships. The topological descriptor Top(G) can also be defined in terms of isomorphisms: Top(G)=Top(H), for every isomorphic graph *H* to *G*. Weiner [6] first developed the notion of topological indices in 1947 while working in the lab on the boiling point of paraffin and referred to it as the path number. The path number was later renamed as the Wiener index.

## 2. Planar Octahedron POH(m) Network Drawing Algorithm

**Figure 1:**
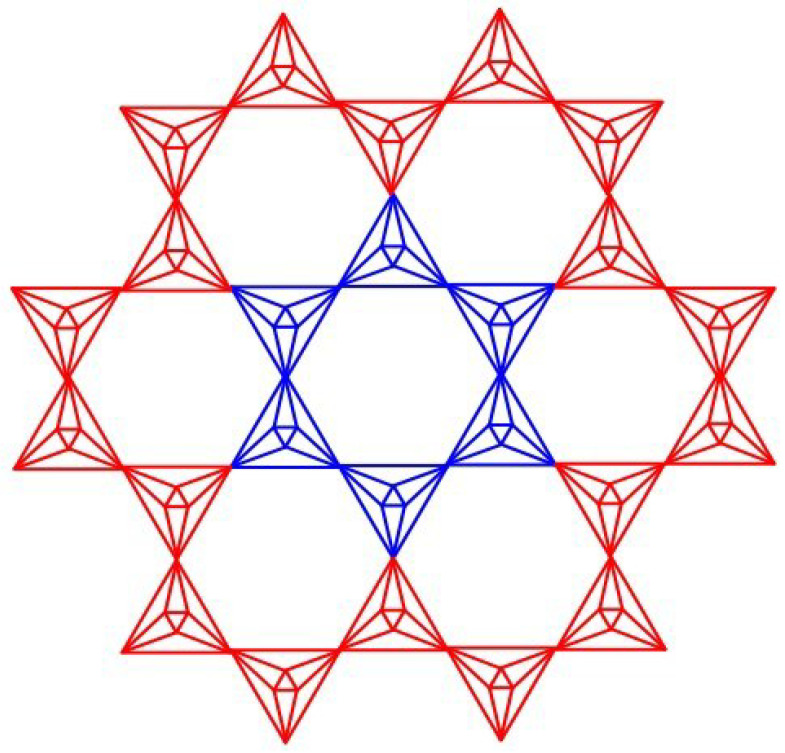
Planar Octahedron network POH(2).

(Step 1:) Draw an *m*-dimensional silicate network [7].
(Step 2:) Each triangle’s centroid should be fixed with new vertices, and those vertices should be connected to the vertices in the corresponding triangle face.
(Step 3:) Connect all of the new centroid vertices on the same silicate sheet.
(Step 4:) Eliminate all silicon vertices. The associated *m*-dimensional graph is known as the planar octahedron network as shown in Figure 1.

In this article, we examined the planar octahedron network and evaluate the eccentricities of the *m* dimension. Let the graph G=G(V,E), where *V* and *E* are non-empty sets of vertices and edges to quantitatively represent molecular processes [8,9,10,11], which is beneficial for researching and using a diverse set of topological indices in theoretical chemistry. In chemical graph theory, there are several topological indices for graphs that are significant in the growth of chemical science.

D(r,s) denotes the distance between *r* and *s* and is defined as the length of the shortest path in *G* if r,s∈V(G). The distance between vertex *r* and the graph’s farthest vertex is defined as the eccentricity. In numerical terms, ϱ(r) = $max_{s\in V(G)}$d(r,s). The total eccentricity index [12] is calculated as follows:

(1) $$\vartheta \left(G\right)=\sum_{c\in V(G)}\varrho \left(c\right),$$

where ϱ(c) is the vertex’s eccentricity [13].

The graph’s average eccentricity *avec*(G) [14] is

(2) $$avec\left(G\right)=\frac{1}{\dot{\iota }}\sum_{c\in V(G)}\varrho \left(c\right),$$

where $\dot{\iota }$ denotes the total number of vertices. Many researchers, including Ilic and Tang [15], have engaged in a few publications to show the average eccentricity index. The eccentricity geometric arithmetic index [16,17,18] is defined by

(3) $$GA_{4}\left(G\right)=\sum_{pc\in E(G)}\frac{2\sqrt{\varrho (p).\varrho (c)}}{\varrho (p)+\varrho (c)}.$$

The eccentricity version of the ABC index [19,20] is denoted as

(4) $$ABC_{5}\left(G\right)=\sum_{pc\in E(G)}\sqrt{\frac{\varrho (p)+\varrho (c)-2}{\varrho (p).\varrho (c)}}.$$

The Zagreb index [21,22,23] comes in the format of

(5) $$ℵ\left(G\right)=\sum_{c\in V(G)}{\left[\varrho \left(c\right)\right]}^{2},$$

(6) $$ℑ\left(G\right)=\sum_{pc\in E(G)}\left[\varrho \left(p\right)\varrho \left(c\right)\right].$$

## 3. Main Results

This section discusses the planar octahedron networks POH(m) for *m* dimensions. The order and size of POH(m) are |V[POH(m)]| = $27m^{2}+m$ and |E[POH(m)]| = $72m^{2}$, where *m* is the dimensions of POH(m).

### Results for Planar Octahedron Network

Planar octahedron networks constructed from honeycomb structures [24,25] are critical in chemistry for researching materials. They have low density and good compression capabilities. These constructions are also used to study stress in a variety of aerospace-related materials. In this section, we computed the eccentricity-based topological indices of a planar octahedron network. The following Table 1 shows the vertex partition of POH(m).

**Theorem** **1.** *For POH(m), ∀m∈N, m≥4, the total eccentricity is equal to*

$$\vartheta \left(POH\left(m\right)\right)=90m^{3}-100m^{2}+138m-10.$$

**Proof.**  Let G≅POH(m), ∀m∈N, and m≥4 be the graph of a planar octahedron network. Through using vertices partitions from Table 1, we determined the total eccentricity index as follows:

$$\vartheta \left(G\right)=\sum_{c\in V(G)}\varrho \left(c\right),$$

$$\begin{array}{ccc} \vartheta (G) & = & \sum_{c\in V_{1}\left(G\right)}\varrho \left(c\right)+\sum_{c\in V_{2}\left(G\right)}\varrho \left(c\right)+\sum_{c\in V_{3}\left(G\right)}\varrho \left(c\right)\\ & & +\sum_{c\in V_{4}\left(G\right)}\varrho \left(c\right), \end{array}$$

$$\begin{array}{ccc} \vartheta (G) & = & \sum_{c=0}^{2}\left(24m+24c-68\right)\left(4m+2c-5\right)\\ & & +\sum_{c=0}^{2}\left(30m+30c-66\right)\left(4m+2c-4\right)\\ & & +\sum_{c=0}^{m-4}\left(20c+4\right)\left(2m+2c+1\right)\\ & & +\sum_{c=0}^{m-4}\left(2m+2c+2\right)\left(34c+24\right), \end{array}$$

$$\begin{array}{ccc} \vartheta (G) & = & \left(24m-66)(4m-5)+(24m-44)(4m-3\right)\\ & & +(24m-20)(4m-1)+(30m-66)(4m-4)\\ & & +(30m-36)(4m-2)+4(30m-6)\\ & & +2m\left(m-3\right)-2m+6+54m{\left(m-3\right)}^{2}\\ & & +18{\left(m-3\right)}^{2}+36{\left(m-3\right)}^{3}, \end{array}$$

After simplification, we obtain

$$\vartheta \left(G\right)=90m^{3}-100m^{2}+138m-10.$$

□

**Theorem** **2.** *For POH(m), ∀m∈N, and m≥4, the average eccentricity index is equal to*

$$avec\left(POH\left(m\right)\right)=\frac{90m^{2}}{27m+1}-\frac{100m}{27m+1}+\frac{138}{27m+1}-\frac{10}{(27m+1)m}.$$

**Proof.**  Let G≅POH(m), ∀m∈N, and m≥4 containing $27m^{2}+m$ vertices and $72m^{2}$ edges. We computed the average eccentricity index as follows by using the vertices division from Table 1:

$$avec\left(G\right)=\frac{1}{\dot{\iota }}\sum_{c\in V(G)}\varrho \left(c\right).$$

$$\begin{array}{ccc} avec(G) & = & \frac{1}{\dot{\iota }}\sum_{c\in V_{1}\left(G\right)}\varrho \left(c\right)+\frac{1}{\dot{\iota }}\sum_{c\in V_{2}\left(G\right)}\varrho \left(c\right)+\frac{1}{\dot{\iota }}\sum_{c\in V_{3}\left(G\right)}\varrho \left(c\right)\\ & & +\frac{1}{\dot{\iota }}\sum_{c\in V_{4}\left(G\right)}\varrho \left(c\right), \end{array}$$

$$\begin{array}{ccc} avec(G) & = & \frac{1}{27m^{2}+m}[\sum_{c=0}^{2}\left(24m+24c-68\right)\left(4m+2c-5\right)\\ & & +\sum_{c=0}^{2}\left(30m+30c-66\right)\left(4m+2c-4\right)\\ & & +\sum_{c=0}^{m-4}\left(20c+4\right)\left(2m+2c+1\right)\\ & & +\sum_{c=0}^{m-4}\left(2m+2c+2\right)\left(34c+24\right)], \end{array}$$

$$\begin{array}{ccc} avec(G) & = & \frac{1}{27m^{2}+m}[\left(24m-66\right)\left(4m-5\right)+\left(24m-44\right)\left(4m-3\right)\\ & & +(24m-20)(4m-1)+(30m-66)(4m-4)\\ & & +(30m-36)(4m-2)+4(30m-6)n\\ & & +2m\left(m-3\right)-2m+6+54m{\left(n-3\right)}^{2}\\ & & +18{\left(n-3\right)}^{2}+36{\left(n-3\right)}^{3}], \end{array}$$

After simplification, we obtain

$$avec\left(G\right)=\frac{90m^{2}}{27m+1}-\frac{100m}{27m+1}+\frac{138}{27m+1}-\frac{10}{(27m+1)m}.$$

□

**Theorem** **3.** *For POH(m), ∀m∈N, and m≥4, the Zagreb eccentricity index is equal to*

$$ℵ\left(POH(m)\right)=306m^{4}+\frac{368}{3}m^{3}-100m^{2}+\frac{742}{3}m-152.$$

**Proof.**  Let G≅POH(m), ∀m∈N, and m≥4 be the planar octahedron network. Using the vertex partitions from Table 1, we obtained the Zagreb eccentricity index as follows:

$$ℵ\left(G\right)=\sum_{s\in \hat{V}\hat{G}}{\left[\varrho \left(c\right)\right]}^{2},$$

$$\begin{array}{ccc} ℵ(G) & = & \sum_{c\in V_{1}\left(G\right)}{\left[\varrho \left(c\right)\right]}^{2}+\sum_{c\in V_{2}\left(G\right)}{\left[\varrho \left(c\right)\right]}^{2}+\sum_{c\in V_{3}\left(G\right)}{\left[\varrho \left(c\right)\right]}^{2}\\ & & +\sum_{c\in V_{4}\left(G\right)}, \end{array}$$

$$\begin{array}{ccc} ℵ(G) & = & \sum_{c=0}^{2}\left(24m+24c-68\right){\left(4m+2c-5\right)}^{2}\\ & & +\sum_{c=0}^{2}\left(30m+30c-66\right){\left(4m+2c-4\right)}^{2}\\ & & +\sum_{c=0}^{m-4}\left(20c+4\right){\left(2m+2c+1\right)}^{2}\\ & & +\sum_{c=0}^{m-4}{\left(2m+2c+2\right)}^{2}\left(34c+24\right), \end{array}$$

$$\begin{array}{ccc} ℵ(G) & = & 14+\left(480m^{3}-96m^{2}\right)+\left(480m^{3}-1056m^{2}+696m-144\right)\\ & & +\left(480m^{3}-2016m^{2}+2592m-1056\right)+\left(384m^{3}-512m^{2}+184m-20\right)\\ & & +\left(384m^{3}-1280m^{2}+1272m-396\right)+\left(384m^{3}-2048m^{2}+3320m-1700\right)\\ & & +\left(4m^{2}-24m+36\right)+\left(\frac{140}{3}m^{3}-420m^{2}+1260m-1260\right)\\ & & +\left(54m^{4}-648m^{3}+2916m^{2}-5832m+4374\right)+\left(4m^{3}-12m^{2}\right)\\ & & +\left(-8m^{2}+24m\right)+\left(108m^{4}-648m^{3}+972m^{2}\right)+(72m^{3}-432m^{2}\\ & & +648m)+\left(144m^{4}-1296m^{3}+3888m^{2}-3888m\right)-\frac{14m}{3}, \end{array}$$

After calculations, we have

$$ℵ\left(G\right)=306m^{4}+\frac{368}{3}m^{3}-100m^{2}+\frac{742}{3}m-152.$$

□

**Theorem** **4.** *For Planar octahedron network POH(m), ∀m∈N, and m≥2, the $GA_{4}$ index of POH(m) is equal to*

$$\begin{array}{ccc} GA_{4}\left(POH\left(m\right)\right) & = & 48\sqrt{2}\left[\sum_{c=0}^{m-2}\left(m-c-1\right)\frac{\sqrt{\left(2m-c-1).(4m-2c-1\right)}}{\left(8m-4c-3\right)}\right]-24m\\ & & +30\left[\sum_{c=0}^{m-1}\left(-c\right)csgn(4m-2c-1)+m(csgn(2m-c-1)\right]\\ & & +24\sqrt{2}\left[\sum_{c=0}^{m-1}\left(5m-5c-2\right)\frac{\sqrt{\left(4m-2c-1).(2m-c\right)}}{\left(8m-4c-1\right)}\right]\\ & & +30\left[\sum_{c=0}^{m-1}\left(m\right)csgn(4m-c)-c(csgn(2m-c)\right]. \end{array}$$

**Proof.**  Let G≅POH(m), ∀m∈N, and m≥2. The eccentricity using the $GA_{4}$ index is calculated as follows using the edge segmentation in Table 2:

$$GA_{4}\left(G\right)=\sum_{pc\in E(G)}\frac{2\sqrt{\varrho (p).\varrho (c)}}{\varrho (p)+\varrho (c)},$$

$$\begin{array}{ccc} GA_{4}\left(G\right) & = & \sum_{pc\in E_{1}\left(G\right)}\frac{2\sqrt{\varrho (p).\varrho (c)}}{\varrho (p)+\varrho (c)}+\sum_{pc\in E_{2}\left(G\right)}\frac{2\sqrt{\varrho (p).\varrho (c)}}{\varrho (p)+\varrho (c)}\\ & & +\sum_{pc\in E_{3}\left(G\right)}\frac{2\sqrt{\varrho (p).\varrho (c)}}{\varrho (p)+\varrho (c)}+\sum_{pc\in E_{4}\left(G\right)}\frac{2\sqrt{\varrho (p).\varrho (c)}}{\varrho (p)+\varrho (c)}, \end{array}$$

$$\begin{array}{ccc} GA_{4}\left(G\right) & = & \sum_{c=0}^{m-2}\left[\left(24m-24c-24\right)\frac{2\sqrt{\left(4m-2c-2).(4m-2c-1\right)}}{\left(4m-2c-2)+(4m-2c-1\right)}\right]\\ & & +\sum_{c=0}^{m-1}\left[\left(30m-30c-24\right)\frac{2\sqrt{\left(4m-2c-1).(4m-2c-1\right)}}{\left(4m-2c-1)+(4m-2c-1\right)}\right]\\ & & +\sum_{c=0}^{m-1}\left[\left(60m-60c-24\right)\frac{2\sqrt{\left(4m-2c-1).(4m-2c\right)}}{\left(4m-2c-1)+(4m-2c\right)}\right]\\ & & +\sum_{c=0}^{m-1}\left[\left(30m-30c\right)\frac{2\sqrt{\left(4m-2c).(4m-2c\right)}}{\left(4m-2c)+(4m-2c\right)}\right], \end{array}$$

$$\begin{array}{ccc} GA_{4}\left(G\right) & = & 24\sum_{c=0}^{m-2}\left[\left(m-c-1\right)\frac{2\sqrt{\left(4m-2c-2).(4m-2c-1\right)}}{\left(4m-2c-2)+(4m-2c-1\right)}\right]\\ & & +6\sum_{c=0}^{m-1}\left[\left(5m-5c-4\right)\frac{2\sqrt{\left(4m-2c-1).(4m-2c-1\right)}}{\left(4m-2c-1)+(4m-2c-1\right)}\right]\\ & & +12\sum_{c=0}^{m-1}\left[\left(5m-5c-2\right)\frac{2\sqrt{\left(4m-2c-1).(4m-2c\right)}}{\left(4m-2c-1)+(4m-2c\right)}\right]\\ & & +30\sum_{c=0}^{m-1}\left[\left(m-c\right)\frac{2\sqrt{\left(4m-2c).(4m-2c\right)}}{\left(4m-2c)+(4m-2c\right)}\right], \end{array}$$

$$\begin{array}{ccc} GA_{4}\left(G\right) & = & 24\sum_{c=0}^{m-2}\left[\left(m-c-1\right)\frac{2\sqrt{2(2m-c-1).(4m-2c-1)}}{2(2m-c-1)+(4m-2c-1)}\right]\\ & & +6\sum_{c=0}^{m-1}\left[\left(5m-5c-4\right)\frac{2\sqrt{\left(4m-2c-1).(4m-2c-1\right)}}{\left(4m-2c-1)+(4m-2c-1\right)}\right]\\ & & +12\sum_{c=0}^{m-1}\left[\left(5m-5c-2\right)\frac{2\sqrt{\left(4m-2c-1).2(2m-c\right)}}{\left(4m-2c-1)+2(2m-c\right)}\right]\\ & & +30\sum_{c=0}^{m-1}\left[\left(m-c\right)\frac{2\sqrt{2(2m-c).2(2m-c)}}{2(m-c)+2(2m-c)}\right], \end{array}$$

After calculation, we obtain the CSGN function, which is used to find the half-plane in which a complex-valued expression or integer *m* residuals are found:

$$\begin{array}{ccc} GA_{4}\left(G\right) & = & 48\sqrt{2}\left[\sum_{c=0}^{m-2}\left(m-c-1\right)\frac{\sqrt{\left(2m-c-1).(4m-2c-1\right)}}{\left(8m-4c-3\right)}\right]-24m\\ & & +30\left[\sum_{c=0}^{m-1}\left(-c\right)csgn(4m-2c-1)+m(csgn(2m-c-1)\right]\\ & & +24\sqrt{2}\left[\sum_{c=0}^{m-1}\left(5m-5c-2\right)\frac{\sqrt{\left(4m-2c-1).(2m-c\right)}}{\left(8m-4c-1\right)}\right]\\ & & +30\left[\sum_{c=0}^{m-1}\left(m\right)csgn(4m-c)-c(csgn(2m-c)\right]. \end{array}$$

□

**Theorem** **5.** *For POH(m), ∀m∈N, and m≥2, the $ABC_{5}$ index of POH(m) is equal to*

$$\begin{array}{ccc} ABC_{5}\left(POH\left(m\right)\right) & = & 12\sqrt{2}\left[\sum_{c=0}^{m-2}\left(m-c-1\right)\sqrt{\frac{\left(8m-4c-5\right)}{\left(2m-c-1)(4m-2c-1\right)}}\right]-24m\\ & & +30\left[\sum_{c=0}^{m-1}\left(-2c\right)\frac{\sqrt{\left(2m-2c-1\right)}}{\left(4m-2c-1\right)}\right]+30\left[\sum_{c=0}^{m-1}\left(2m\right)\frac{\sqrt{\left(2m-2c-1\right)}}{\left(4m-2c-1\right)}\right]\\ & & +6\sqrt{2}\left[\sum_{c=0}^{m-1}\left(5m-5c-2\right)\sqrt{\frac{\left(8m-4c-3\right)}{\left(4m-2c-1).(2m-c\right)}}\right]\\ & & +15\sqrt{2}\left[\sum_{c=0}^{m-1}m\left(\frac{\sqrt{\left(4m-2c-1\right)}}{\left(2m-c\right)}\right)\right]-15\sqrt{2}\left[\sum_{c=0}^{m-1}c\left(\frac{\sqrt{\left(4m-2c-1\right)}}{\left(2m-c\right)}\right)\right]. \end{array}$$

**Proof.**  Let G≅POH(m), ∀m∈N, and m≥2 be the graph planar octahedron network. Applying the edge partition in Table 2, we estimated the eccentricity $ABC_{5}$ as follows:

$$ABC_{5}\left(G\right)=\sum_{pc\in E(G)}\sqrt{\frac{\varrho (p)+\varrho (c)-2}{\varrho (p).\varrho (c)}},$$

$$\begin{array}{ccc} ABC_{5}\left(G\right) & = & \sum_{pc\in E_{1}\left(G\right)}\sqrt{\frac{\varrho (p)+\varrho (c)-2}{\varrho (p).\varrho (c)}}+\sum_{pc\in E_{2}\left(G\right)}\sqrt{\frac{\varrho (p)+\varrho (c)-2}{\varrho (p).\varrho (c)}}\\ & & +\sum_{pc\in E_{3}\left(G\right)}\sqrt{\frac{\varrho (p)+\varrho (c)-2}{\varrho (p).\varrho (c)}}+\sum_{pc\in E_{4}\left(G\right)}\sqrt{\frac{\varrho (p)+\varrho (c)-2}{\varrho (p).\varrho (c)}}, \end{array}$$

$$\begin{array}{ccc} ABC_{5}\left(G\right) & = & \sum_{c=0}^{m-2}\left[\left(24m-24c-24\right)\sqrt{\frac{(4m-2c-2)+(4m-2c-1)-2}{\left(4m-2c-2).(4m-2c-1\right)}}\right]\\ & & +\sum_{c=0}^{m-1}\left[\left(30m-30c-24\right)\sqrt{\frac{(4m-2c-1)+(4m-2c-1)-2}{\left(4m-2c-1).(4m-2c-1\right)}}\right]\\ & & +\sum_{c=0}^{m-1}\left[\left(60m-60c-24\right)\sqrt{\frac{(4m-2c-1)+(4m-2c)-2}{\left(4m-2c-1).(4m-2c\right)}}\right]\\ & & +\sum_{c=0}^{m-1}\left[\left(30m-30c\right)\sqrt{\frac{(4m-2c)+(4m-2c)-2}{\left(4m-2c).(4m-2c\right)}}\right], \end{array}$$

$$\begin{array}{ccc} ABC_{5}\left(G\right) & = & 12\sqrt{2}\left[\sum_{c=0}^{m-2}\left(m-c-1\right)\sqrt{\frac{\left(8m-4c-5\right)}{\left(2m-c-1)(4m-2c-1\right)}}\right]\\ & & -24m+30[\sum_{c=0}^{m-1}\left(-c\right)\left(2\sqrt{\frac{\left(2m-2c-1\right)}{\left(4m-2c-1).(4m-2c-1\right)}}\right)+\\ & & m\left(2\sqrt{\frac{\left(2m-2c-1\right)}{\left(4m-2c-1).(4m-2c-1\right)}}\right)]+6\sqrt{2}[\sum_{c=0}^{m-1}(5m-5c\\ & & -2)\sqrt{\frac{\left(8m-4c-3\right)}{\left(4m-2c-1).(2m-c\right)}}]+30[\sum_{c=0}^{m-1}(m(\frac{\sqrt{2}}{2}\\ & & \sqrt{\frac{\left(4m-2c-1\right)}{\left(2m-c).(2m-c\right)}}))-c\left(\frac{\sqrt{2}}{2}\sqrt{\frac{\left(4m-2c-1\right)}{\left(2m-c).(2m-c\right)}}\right)], \end{array}$$

$$\begin{array}{ccc} ABC_{5}\left(G\right) & = & 12\sqrt{2}\left[\sum_{c=0}^{m-2}\left(m-c-1\right)\sqrt{\frac{\left(8m-4c-5\right)}{\left(2m-c-1)(4m-2c-1\right)}}\right]-24m\\ & & +30\left[\sum_{c=0}^{m-1}\left(-2c\right)\sqrt{\frac{\left(2m-2c-1\right)}{{\left(4m-2c-1\right)}^{2}}}\right]+30\left[\sum_{c=0}^{m-1}\left(2m\right)\sqrt{\frac{\left(2m-2c-1\right)}{{\left(4m-2c-1\right)}^{2}}}\right]\\ & & +6\sqrt{2}\left[\sum_{c=0}^{m-1}\left(5m-5c-2\right)\sqrt{\frac{\left(8m-4c-3\right)}{\left(4m-2c-1).(2m-c\right)}}\right]+30\\ & & \left[\sum_{c=0}^{m-1}m\left(\frac{\sqrt{2}}{2}\sqrt{\frac{\left(4m-2c-1\right)}{{\left(2m-c\right)}^{2}}}\right)\right]-30\left[\sum_{c=0}^{m-1}c\left(\frac{\sqrt{2}}{2}\sqrt{\frac{\left(4m-2c-1\right)}{{\left(2m-c\right)}^{2}}}\right)\right], \end{array}$$

$$\begin{array}{ccc} ABC_{5}\left(G\right) & = & 12\sqrt{2}\left[\sum_{c=0}^{m-2}\left(m-c-1\right)\sqrt{\frac{\left(8m-4c-5\right)}{\left(2m-c-1)(4m-2c-1\right)}}\right]-24m\\ & & +30\left[\sum_{c=0}^{m-1}\left(-2c\right)\frac{\sqrt{\left(2m-2c-1\right)}}{\left(4m-2c-1\right)}\right]+30\left[\sum_{c=0}^{m-1}\left(2m\right)\frac{\sqrt{\left(2m-2c-1\right)}}{\left(4m-2c-1\right)}\right]\\ & & +6\sqrt{2}\left[\sum_{c=0}^{m-1}\left(5m-5c-2\right)\sqrt{\frac{\left(8m-4c-3\right)}{\left(4m-2c-1).(2m-c\right)}}\right]+30\\ & & \left[\sum_{c=0}^{m-1}m\left(\frac{\sqrt{2}}{2}\frac{\sqrt{\left(4m-2c-1\right)}}{\left(2m-c\right)}\right)\right]-30\left[\sum_{c=0}^{m-1}c\left(\frac{\sqrt{2}}{2}\frac{\sqrt{\left(4m-2c-1\right)}}{\left(2m-c\right)}\right)\right], \end{array}$$

$$\begin{array}{ccc} ABC_{5}\left(G\right) & = & 12\sqrt{2}\left[\sum_{c=0}^{m-2}\left(m-c-1\right)\sqrt{\frac{\left(8m-4c-5\right)}{\left(2m-c-1)(4m-2c-1\right)}}\right]-24m\\ & & +30\left[\sum_{c=0}^{m-1}\left(-2c\right)\frac{\sqrt{\left(2m-2c-1\right)}}{\left(4m-2c-1\right)}\right]+30\left[\sum_{c=0}^{m-1}\left(2\right)\frac{\sqrt{\left(2m-2c-1\right)}}{\left(4m-2c-1\right)}\right]\\ & & +6\sqrt{2}\left[\sum_{c=0}^{m-1}\left(5m-5c-2\right)\sqrt{\frac{\left(8m-4c-3\right)}{\left(4m-2c-1).(2m-c\right)}}\right]+30\frac{\sqrt{2}}{2}\\ & & \left[\sum_{c=0}^{m-1}m\left(\frac{\sqrt{\left(4m-2c-1\right)}}{\left(2m-c\right)}\right)\right]-30\frac{\sqrt{2}}{2}\left[\sum_{c=0}^{m-1}c\left(\frac{\sqrt{\left(4m-2c-1\right)}}{\left(2m-c\right)}\right)\right], \end{array}$$

after calculation, we have

$$\begin{array}{ccc} ABC_{5}\left(G\right) & = & 12\sqrt{2}\left[\sum_{c=0}^{m-2}\left(m-c-1\right)\sqrt{\frac{\left(8m-4c-5\right)}{\left(2m-c-1)(4m-2c-1\right)}}\right]-24m\\ & & +30\left[\sum_{c=0}^{m-1}\left(-2c\right)\frac{\sqrt{\left(2m-2c-1\right)}}{\left(4m-2c-1\right)}\right]+30\left[\sum_{c=0}^{m-1}\left(2m\right)\frac{\sqrt{\left(2m-2c-1\right)}}{\left(4m-2c-1\right)}\right]\\ & & +6\sqrt{2}\left[\sum_{c=0}^{m-1}\left(5m-5c-2\right)\sqrt{\frac{\left(8m-4c-3\right)}{\left(4m-2c-1).(2m-c\right)}}\right]+15\sqrt{2}\\ & & \left[\sum_{c=0}^{m-1}m\left(\frac{\sqrt{\left(4m-2c-1\right)}}{\left(2m-c\right)}\right)\right]-15\sqrt{2}\left[\sum_{c=0}^{m-1}c\left(\frac{\sqrt{\left(4m-2c-1\right)}}{\left(2m-c\right)}\right)\right]. \end{array}$$

□

**Theorem** **6.** *For POH(m), ∀m∈N, and m≥2, ℑ(G) of POH(m) is equal to*

$$ℑ\left(POH(m)\right)=816m^{4}+160m^{3}-9m^{2}-m.$$

**Proof.**  Let G≅POH(m), ∀m∈N, and m≥2 be the planar octahedron network. Utilizing the edge partition in Table 2, we derived the third Zagreb eccentricity index as follows:

$$ℑ\left(G\right)=\sum_{pc\in E(G)}\left[\varrho \left(p\right)\varrho \left(c\right)\right],$$

$$\begin{array}{ccc} ℑ(G) & = & \sum_{pc\in E_{1}\left(G\right)}\left[\varrho \left(p\right)\varrho \left(c\right)\right]+\sum_{pc\in E_{2}\left(G\right)}\left[\varrho \left(p\right)\varrho \left(c\right)\right]\\ & & +\sum_{pc\in E_{3}\left(G\right)}\left[\varrho \left(p\right)\varrho \left(c\right)\right]+\sum_{pc\in E_{4}\left(G\right)}\left[\varrho \left(p\right)\varrho \left(c\right)\right], \end{array}$$

$$\begin{array}{ccc} ℑ(G) & = & \sum_{c=0}^{m-2}\left(24m-24c-24\right)\left[(4m-2c-2)(4m-2c-1)\right]\\ & & +\sum_{c=0}^{m-1}\left(30m-30c-24\right)\left[(4m-2c-1)(4m-2c-1)\right]\\ & & +\sum_{c=0}^{m-1}\left(60m-60c-24\right)\left[(4m-2c-1)(4m-2c)\right]\\ & & +\sum_{c=0}^{m-1}\left(30m-30c\right)\left[(4m-2c)(4m-2c)\right], \end{array}$$

$$\begin{array}{ccc} ℑ(G) & = & (384m^{3}\left(m-1\right)-384m^{2}{\left(m-1\right)}^{2}+160m{\left(m-1\right)}^{3}-24{\left(m-1\right)}^{4}\\ & & -288m^{2}\left(m-1\right)+168m{\left(m-1\right)}^{2}-32{\left(n-1\right)}^{3}+8m\left(m-1\right)\\ & & +11m-8+170m^{4}-84m^{3}-35m^{2}+[\sum_{c=0}^{m-1}\left(60m-1\left(60c\right)-24\right)\\ & & \left(4m-1\left(2c\right)-1\right)\left(4m-1\left(2c\right)\right)]+[\sum_{c=0}^{m-1}\left(30m-1\left(30c\right)\right)\left(4m-1\left(2c\right)\right)\\ & & (4m-1(2c))], \end{array}$$

$$\begin{array}{ccc} ℑ(G) & = & (384m^{3}\left(m-1\right)-384m^{2}{\left(m-1\right)}^{2}+160m{\left(m-1\right)}^{3}-24{\left(m-1\right)}^{4}\\ & & -288m^{2}\left(m-1\right)+168m{\left(m-1\right)}^{2}-32{\left(m-1\right)}^{3}+8m\left(m-1\right)-m\\ & & -8+680m^{4}+312m^{3}-17m^{2}, \end{array}$$

$$\begin{array}{ccc} ℑ(G) & = & 384m^{4}-384m^{3}+\left(-384m^{4}+768m^{3}-384m^{2}\right)+(160m^{4}-480m^{3}\\ & & +480m^{2}-160m)+\left(-24m^{4}+96m^{3}-144m^{2}+96m-24\right)+(-288m^{3}\\ & & +288m^{2}+\left(168m^{3}-336m^{2}+168m\right)+\left(-32m^{3}+96m^{2}-96m+32\right)\\ & & +\left(8m^{2}-8m\right)-m-8+680m^{4}+312m^{3}-17m^{2}, \end{array}$$

$$\begin{array}{ccc} ℑ(G) & = & \left(384m^{4}-384m^{4}+160m^{4}-24m^{4}+680m^{4}\right)+(-384m^{3}+768m^{3}\\ & & -480m^{3}+96m^{3}-288m^{3}-32m^{3}+312m^{3})+(384m^{2}+480m^{2}\\ & & -144m^{2}+288m^{2}-336m^{2}+96m^{2}+8m^{2}-17m^{2})+(-160m\\ & & +96m+168m-96m-8m-m)+(-24+32-8), \end{array}$$

after calculation, we have

$$ℑ\left(G\right)=816m^{4}+160m^{3}-9m^{2}-m.$$

□

## 4. Conclusions

In this article, we have calculated eccentricity-based topological indices, namely, total eccentricity, average eccentricity, and geometric arithmetic $\left(GA_{4}\right)$, as well as atom bond connectivity $\left(ABC_{5}\right)$ for the planar octahedron network POH(m). Octahedron networks have a variety of useful applications in pharmaceuticals, electronics, and networking. Individuals working in computer science and chemistry may find these findings beneficial from a chemical point of view. There are a number of unsolved problems in the evaluation of associated derived networks.

## Figures and Tables

**Table 1:** Vertex Partition of POH(m) with respect to Eccentricities.

Sets	ϱ(c)	Vertices	Range
$V_{1}$	(4m−5+2c)	24m−68+24c	0≤c≤2
$V_{2}$	(4m−4+2c)	30m−66+30c	0≤c≤2
$V_{3}$	(2m+1+2c)	4+20c	0≤c≤m−4
$V_{4}$	(2m+2+2c)	24+34c	0≤c≤m−4

**Table 2:** Edge Partition of POH(m).

Sets	ϱ(p),ϱ(c)	Edges	Range
$E_{1}$	(4m−2c−2),(4m−2c−1)	24m−24c−24	0≤c≤m−2
$E_{2}$	(4m−2c−1),(4m−2c−1)	30m−30c−24	0≤c≤m−1
$E_{3}$	(4m−2c−1),(4m−2c)	60m−60c−24	0≤c≤m−1
$E_{4}$	(4m−2c),(4m−2c)	30m−30c	0≤c≤m−1

## Data Availability

No data available to support this study.
